# Congenital Pouch Colon with Double Meckel’s Diverticulum in a Patient with Persistent Cloaca 

**Published:** 2015-01-10

**Authors:** Nand Kishor Shinde, Praveen Jhanwar, Nitin Pant, Subhasis Roy Choudhury, Rajiv Chadha

**Affiliations:** The Department of Pediatric Surgery, Lady Hardinge Medical College and Kalawati Saran Children’s Hospital, New Delhi-110018, INDIA

**Dear Sir**

A 5-day-old girl was brought with complaints of vomiting and non-passage of urine and stools for one day. The child had absent anal orifice and was passing urine and stools from a single perineal opening. Examination showed clinical evidence of sepsis and a grossly distended, tense, tympanitic, and tender abdomen. The perineum was well developed and the sacrum was normal on palpation. X-ray abdomen showed a massive pneumoperitoneum. After optimization, exploratory laparotomy revealed a Type II congenital pouch colon (CPC), with 3cm of normal proximal colon, a long appendix, and a large perforation of the colonic pouch near its center on anterior aspect. There were 2 broad-based diverticulae at the anti-mesenteric side of the terminal ileum at an interval of 3 cm, the distal diverticulum being 3 cm proximal to the ileocecal junction (ICJ) (Fig. 1) .The perforation in the colonic pouch was closed in 2 layers and a divided distal ileostomy was constructed proximal to the colonic pouch. The child is at present awaiting further corrective surgery. A relatively high incidence of occurrence of a Meckel’s diverticulum in association with CPC has been reported in the literature [1,2] . The presence of two Meckel’s diverticulae in a patient of CPC has been reported twice earlier [1, 3]. Mathur et al [3] described a boy with Type I CPC and two Meckel’s diverticulae, situated 40 cm and 50 cm respectively proximal to the ICJ. In contrast, in our patient, the 2 diverticulae were situated 3 cm and 6 cm proximal to the ICJ. Significantly, an earlier report described a boy with Type II CPC and a Meckel’s diverticulum located just 3-5 cm proximal to the ICJ indicating that some cases of Types I/ II CPC may have shortening of the bowel distal to the omphalomesenteric duct [4]. The additional frequent occurrence of appendiceal abnormalities including agenesis or duplication in Types I/ II CPC therefore suggests that the abnormal embryogenesis in these cases may involve the entire post-arterial limb of the midgut as well as the hindgut.

**Figure:**
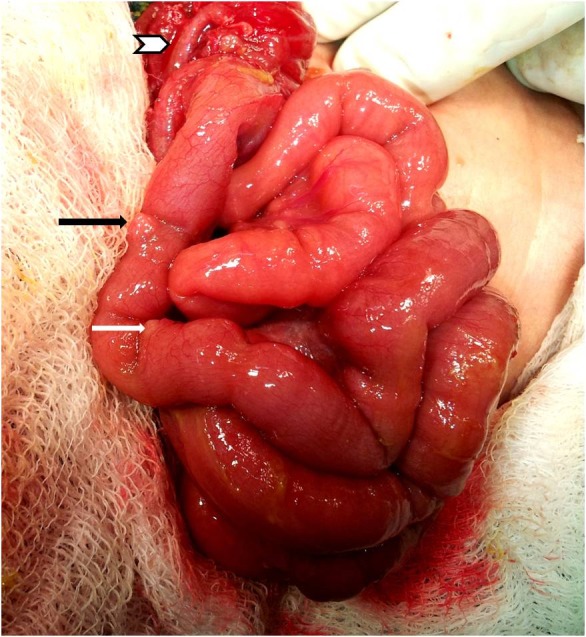
Figure 1: Operative photograph showing the two Meckel’s diverticulae (arrows) and the long appendix (chevron).

## Footnotes

**Source of Support:** Nil

**Conflict of Interest:** None

